# Adaptive Pathways: Possible Next Steps for Payers in Preparation for Their Potential Implementation

**DOI:** 10.3389/fphar.2017.00497

**Published:** 2017-08-23

**Authors:** Patricia Vella Bonanno, Michael Ermisch, Brian Godman, Antony P. Martin, Jesper Van Den Bergh, Liudmila Bezmelnitsyna, Anna Bucsics, Francis Arickx, Alexander Bybau, Tomasz Bochenek, Marc van de Casteele, Eduardo Diogene, Irene Eriksson, Jurij Fürst, Mohamed Gad, Ieva Greičiūtė-Kuprijanov, Martin van der Graaff, Jolanta Gulbinovic, Jan Jones, Roberta Joppi, Marija Kalaba, Ott Laius, Irene Langner, Ileana Mardare, Vanda Markovic-Pekovic, Einar Magnusson, Oyvind Melien, Dmitry O. Meshkov, Guenka I. Petrova, Gisbert Selke, Catherine Sermet, Steven Simoens, Ad Schuurman, Ricardo Ramos, Jorge Rodrigues, Corinne Zara, Eva Zebedin-Brandl, Alan Haycox

**Affiliations:** ^1^Department of Pharmacoepidemiology, Strathclyde Institute of Pharmacy and Biomedical Sciences, University of Strathclyde Glasgow, United Kingdom; ^2^Pharmaceutical Department, National Association of Statutory Health Insurance Funds Berlin, Germany; ^3^Health Economics Centre, University of Liverpool Management School Liverpool, United Kingdom; ^4^Division of Clinical Pharmacology, Karolinska Institutet Stockholm, Sweden; ^5^Department of Health Ecorys, Rotterdam, Netherlands; ^6^National Research Institution for Public Health Moscow, Russia; ^7^Mechanism of Coordinated Access to Orphan Medicinal Products (MoCA) Brussels, Belgium; ^8^Department of Pharmaceutical Policy, National Institute for Health and Disability Insurance Bruxelles, Belgium; ^9^Zilveren Kruis Achmea Leiden, Netherlands; ^10^Department of Drug Management, Faculty of Health Sciences, Jagiellonian University Medical College Kraków, Poland; ^11^Clinical Pharmacology Service, University Hospital Vall d'Hebron, Universitat Autonoma de Barcelona Barcelona, Spain; ^12^Department of Healthcare Development, Stockholm County Council Stockholm, Sweden; ^13^Department of Medicine Solna, Karolinska Institutet Stockholm, Sweden; ^14^Medicinal Products Department, Health Insurance Institute of Slovenia Ljubljana, Slovenia; ^15^Global Health and Development Group, Imperial College London, United Kingdom; ^16^Department of Pharmacy, Ministry of Health of the Republic of Lithuania Vilnius, Lithuania; ^17^National Health Care Institute (ZIN) Diemen, Netherlands; ^18^Department of Pathology, Forensic Medicine and Pharmacology, Faculty of Medicine, Vilnius University Vilnius, Lithuania; ^19^State Medicines Control Agency Vilnius, Lithuania; ^20^Scottish Medicines Consortium Glasgow, United Kingdom; ^21^Clinical Research and Drug Assessment Unit Verona, Italy; ^22^Pediatric Cardiology, Primary Healthcare Centre “Zemun” Belgrade, Serbia; ^23^Department of Post-authorisation Safety, State Agency of Medicines Tartu, Estonia; ^24^Wissenschaftliches Institut der AOK Berlin, Germany; ^25^Faculty of Medicine, Public Health and Management Department, “Carol Davila” University of Medicine and Pharmacy Bucharest Bucharest, Romania; ^26^Ministry of Health and Social Welfare Banja Luka, Bosnia and Herzegovina; ^27^Department of Social Pharmacy, Medical Faculty, University of Banja Luka Banja Luka, Bosnia and Herzegovina; ^28^Department of Health Services, Ministry of Health Reykjavík, Iceland; ^29^Norwegian Directorate for Health Oslo, Norway; ^30^Department of Social Pharmacy and Pharmacoeconomics, Faculty of Pharmacy, Medical University of Sofia Sofia, Bulgaria; ^31^Institut de Recherche et Documentation en Economie de la Santé (IRDES) Paris, France; ^32^KU Leuven Department of Pharmaceutical and Pharmacological Sciences Leuven, Belgium; ^33^Health Technology Assessment, Pricing and Reimbursement Department, Central Administration of the Health System, National Authority of Medicines and Health Products (I.P., INFARMED) Lisboa, Portugal; ^34^Barcelona Health Region, Catalan Health Service Barcelona, Spain; ^35^Department of Pharmaceutical Affairs, Main Association of Austrian Social Insurance Institutions Vienna, Austria

**Keywords:** European Medicines Agency, Adaptive Pathways, Health Technology Assessment, marketing authorization, payers

## Abstract

Medicines receiving a conditional marketing authorization through Medicines Adaptive Pathways to Patients (MAPPs) will be a challenge for payers. The “introduction” of MAPPs is already seen by the European Medicines Agency (EMA) as a fait accompli, with payers not consulted or involved. However, once medicines are approved through MAPPs, they will be evaluated for funding by payers through different activities. These include Health Technology Assessment (HTA) with often immature clinical data and high uncertainty, financial considerations, and negotiations through different types of agreements, which can require monitoring post launch. Payers have experience with new medicines approved through conditional approval, and the fact that MAPPs present additional challenges is a concern from their perspective. There may be some activities where payers can collaborate. The final decisions on whether to reimburse a new medicine via MAPPs will have more variation than for medicines licensed via conventional processes. This is due not only to increasing uncertainty associated with medicines authorized through MAPPs but also differences in legal frameworks between member states. Moreover, if the financial and side-effect burden from the period of conditional approval until granting full marketing authorization is shifted to the post-authorization phase, payers may have to bear such burdens. Collection of robust data during routine clinical use is challenging along with high prices for new medicines during data collection. This paper presents the concept of MAPPs and possible challenges. Concerns and potential ways forward are discussed and a number of recommendations are presented from the perspective of payers.

## Introduction

Fifty years from the introduction of Council Directive 65/65/EEC^1^, there seems to be consensus from different stakeholders that despite considerable achievements, the pharmaceutical framework still has room for improvement. Existing gaps include a lack of the full achievement of the objectives as set out by the Treaties, problems with the availability and affordability of medicines for all citizens of the European Union (EU), the need for incentives for innovation, and the need for increased development of pathways for access to medicines for rare diseases that address unmet medical need (Heads of Medicines Agencies, 2007; Commission of the European Communities, 2008; Matrix Insight, 2012; Council of the EU, 2016).

Minimizing health care disparities is fundamental for the equitable and progressive achievement of universal health coverage. There are concerns that the rate at which new medicines are introduced may vary, in particular that new treatments may be taken up less quickly and in lower numbers in certain countries due to challenges in access, availability and affordability (Putrik et al., 2014; UK, 2016). Equity is an ethical concept which is based on the principle of distributive justice and refers to social justice or fairness (Rawls, 1971; Sen, 2010). Equity in health can largely be defined as the absence of systematic disparities in health between social groups who have different levels of underlying social advantage or different positions in social hierarchy (Whitehead, 1991; Culyer, 1993; Oliver, 2004; Hosseinpoor et al., 2014).

Access to medicines in therapeutic areas with unmet medical need typically has a high public health value. Over the years, there have been various legislative and non-legislative initiatives to address this issue. The European Medicines Agency (EMA) included the objective of earlier access to medicines for unmet medical need in its priorities, and in recent years has proposed “Adaptive Pathways”, also known as Medicines Adaptive Pathways to Patients (MAPPs), as a key initiative to address this. Adaptive Pathways are presented as an approach intended to maximize the positive impact of new medicines on public health, achieved by balancing the need for timely patient access through staggered approval with iterative phases by using existing tools combined with a more flexible use of the existing regulatory framework (Eichler et al., 2012, 2013, 2015; European Medicines Agency, 2014; Rasi and Eicher, 2016).

Through Adaptive Pathways, a medicine can follow an “iterative” process whereby it can initially be approved only for a smaller group of patients (based on limited scientific evidence) and then, when more evidence is gathered, the medicine will be more widely approved. Moreover, a medicine could be approved on the basis of surrogate endpoints, which would need to be verified later with more clinically important outcome endpoints (Ermisch et al., 2016).

Payers support innovation and access to new effective medicines, especially where there is true unmet medical need, as illustrated by initiatives in the UK to accelerate access to new innovative medicines (UK, 2016). In practice though, only a limited number of new medicines are truly innovative and address real gaps in treatment (Garattini et al., 2008; Ermisch et al., 2016; Prescrire Editorial, 2016). As a result, and when combined with the many challenges involved, payers and others have concerns with the concept of Adaptive Pathways as proposed by the EMA. These concerns have been summarized and published in a series of papers (Banzi et al., 2015; Joint Briefing Paper, 2015; Joint response to EMA public consultation, 2015; Cattarin, 2016; Davis et al., 2016; Ermisch et al., 2016; Garattini et al., 2016; Hawkes, 2016a,b; Natsis, 2016). These concerns were aggravated by the lack of clear justification for the need of Adaptive Pathways over and above the available regulatory processes (Joint Briefing Paper, 2015; Ermisch et al., 2016; Joint Press Release, 2016).

A major step in the MAPPs initiative was the publication of the “Final report on the adaptive pathways pilot” (European Medicines Agency, 2016). This report showed that in spite of the concerns expressed by key stakeholders, the EMA was pushing forward with the MAPPs concept. Issues identified for further reflection included a proposal for the involvement of all key stakeholders [patients, healthcare professionals, Health Technology Assessment (HTA) bodies and payers]. EMA “expected” the various stakeholders to cooperate to ensure timely and affordable access to new innovative medicines. Unfortunately, payers were not involved in the process of Adaptive Pathways and, in the report of the pilot, the EMA only considered to invite payers to come on board “if relevant”. At this stage, the EMA only considered possible “fine-tuning” of the concept. In next steps, the possible incorporation of Adaptive Pathways into existing platforms for parallel regulatory-HTA scientific advice was to be investigated (European Medicines Agency, 2016).

As a result, payers need to plan and be prepared for evaluation of medicinal products with a European conditional marketing authorization through the Adaptive Pathways concept especially if higher prices are requested vs. current standards. As the concept of Adaptive Pathways was an initiative to improve access to medicines for unmet medical need, it will be important to measure the level of the true achievement of this objective in the future. This is a prerequisite to allow any considerations by payers.

The objective of this paper is to consider possible next steps and alternatives for payers in preparation for the potential implementation of the concept of Adaptive Pathways.

## The pharmaceutical framework in europe

The pharmaceutical framework encompasses a complex real-life set of systems.

It consists of a series of parallel and consecutive systems, which although independent in structure and function, are highly influential on each other. These systems differ in order and function between Member States but generally follow similar activities. These include research and development, evaluation and marketing authorization, post-authorization monitoring, setting of prices, manufacture and supply, HTA and reimbursement, procurement, provision of health services, prescribing and dispensing, administration, and use of medicines. Within the confines of the current legal structure, the pharmaceutical framework is constantly evolving through the initiatives of the different stakeholders who set policies, introduce activities and build structures for their functioning within the pharmaceutical framework. The final outcomes of the framework depend on the outcomes of the individual systems and also on the logical flow from one system to another, which can be blocked at any system within the framework, affecting the systems which follow it and also possibly those which come before it (Vella Bonanno, 2003, 2010).

## Framework, initiatives and tools supporting the early access of medicinal products

### Legal and governance structure

The Treaties of the EU set the mandate for the structure and systems of the pharmaceutical framework, including the competences and responsibilities of the EU and the Member States and the balances between them. The main objective of the framework is a high level of protection of human health and the improvement of public health. The Treaties clarify principles including the principle of subsidiarity and the principle of conferral. The principle of subsidiarity determines when the EU is competent to legislate, while the principle of conferral states that the EU shall act within the limits of the competences conferred to it by the Member States in the Treaties, and that the competences not conferred upon the Union by the Treaties remain within the Member States^2^. The European Commission (EC), the European Parliament, and the Council can adopt measures for setting high standards of quality and safety for medicinal products (Council of the EU, 2016).

The main objective of European medicines regulation is to ensure that available medicines are of good quality, safe and efficacious. The bodies responsible for the regulation of medicinal products consist of the network of the EMA and the regulatory agencies in the individual member states. The EMA is governed by Regulation (EC) No. 726/2004^3^. The responsibility for the regulatory benefit/risk governance of medicinal products authorized through the centralized procedure lies with the EC. The responsibility for the marketing authorization of medicinal products receiving a national marketing authorization lies with the licensing authority within the respective Member State. The number of medicinal products approved through the centralized procedure which are truly innovative is limited (Motola et al., 2006).

There have been a number of updates to the pharmaceutical legislation addressing unmet medical need. There were specific changes to the legislation for centrally authorized products (Regulation (EC) No. 726/2004) which allow for early access of new medicinal products including conditional approval [Article 14 (7)] and compassionate use (Article 83). Conditional marketing authorization is part of the marketing authorization decision and appreciably affects the post-authorization phase. In specific situations of unmet medical need, and in the interest of public health, the legislation provides the possibility for granting conditional marketing authorizations on the basis of less complete data than is required for a normal submission. This still requires the assumption that the benefit/risk profile is positive and that knowledge gaps will be closed. Following conditional approval, the pertinent medicines should be subject to obligations of fulfillment of the data requirements. The marketing authorization is not meant to remain conditional indefinitely (Regulation (EC) No. 507/2006)^4^. To date, the experience with conditional approval has been modest. From 2006 to June 2014, 26 products were granted a conditional marketing authorization and a number of these have not fulfilled their post-authorization obligations (Banzi et al., 2015). A more recent update published by the EMA reported that 30 products were approved through conditional approval from 2006 to 30th June 2016. By the date of publication in early 2017, eleven of these approvals were converted into standard marketing authorizations, two were withdrawn for commercial reasons and seventeen were still conditional. Of the conditional marketing authorizations, none were authorized for more than 5 years (European Medicines Agency, 2017).

Compassionate use is an initiative which takes place prior to marketing authorization. This initiative provides for the use of a product eligible under the centralized procedure to be available to a group of patients “*with chronically or severely debilitating disease or whose disease is considered to be life-threatening and who cannot be treated satisfactorily by an authorized medicinal product”*. The medicinal product concerned must either be considered for authorization in accordance with the centralized procedure or must be undergoing clinical trials. Regulation (EC) No. 141/2000^5^ on orphan medicinal products and Regulation (EC) No 1901/2006^6^ on paediatric medicines were aimed at supporting research and development for life-threatening and rare diseases and for indications in children respectively. New pharmacovigilance legislation came into force in 2012 and provides for monitoring of safety aspects throughout the medicine's life-cycle (Regulation (EU) No. 1235/2010)^7^.

### Pricing and reimbursement of medicinal products

The competence and responsibility for decisions regarding which medicinal products are reimbursed and at what price, lie with the Member States (Council of the EU, 2016). The whole procedure is regulated by the “Transparency Directive” (Council Directive 89/105/EEC). A marketing authorization gives the right to the marketing authorization holder (MAH) to submit an application for a marketing authorization in countries with a positive list, and have this application evaluated within 90 to 180 days (Article 6).

National pharmaceutical reimbursement systems are governed by national legislation and policies, which cover mainly aspects which are of national jurisdiction such as pricing and reimbursement procedures, national essential medicines lists, medicine financing, and human resources related to pharmaceutical activities. National stakeholders of the pharmaceutical framework include Drugs and Therapeutics Committees which can include different experts in the field, policy makers, operators of the supply chain, health care professionals and patients, and patient organizations (WHO, 2001). Certain Central and Eastern European countries currently experience significant delays in the introduction of new expensive innovative medicines on their positive medicines lists. In some countries, marketing authorization holders are not interested in launching certain products in less favorable and attractive markets possibly due to economies of scale while another reason for delays in the launch of new medicines on national markets are stringent price controls alongside external reference pricing (Heads of Medicines Agencies, 2007; Leopold et al., 2012; Dimitrova et al., 2013; Kamusheva et al., 2013).

Member States differ in the criteria and the considerations which are used in the evaluation of new medicines such as the way in which they deal with off-label comparators, subgroup analyses and the role of cost-effectiveness. These include different requirements for comparators, and whether health economic techniques are used in reimbursement decision making such as cost/ QALY (quality adjusted life year), with or without threshold levels; alternatively, assigning a level of innovation in preparation for pricing discussions, and the ability to restrict patients to defined sub-groups for funding with or without managed entry agreements (Ferrario and Kanavos, 2013; Godman et al., 2013b, 2016b,a; Malmstrom et al., 2013; Paris and Belloni, 2013; Matusewicz et al., 2015; WHO, 2015).

Overall, accessibility and funding of new medicines does vary among Member States, as seen with the anti-TNF alpha medicines for rheumatoid arthritis and Crohn's disease and new treatments for hepatitis C, depending on available resources. Similarly for existing medicines such as the proton-pump inhibitors and statins with different patient co-payments and prescribing restrictions between countries (Godman et al., 2010; Putrik et al., 2014; de Bruijn et al., 2016; Garuoliene et al., 2016; Kostic et al., 2017).

Consequently, there is no guarantee that marketing authorization through the process of Adaptive Pathways will result in earlier access to medicines in all Member States. These delays may result in disparity of burden sharing and impact on the implementation of Adaptive Pathways among the different European countries.

As stated, currently there are different mechanisms for pricing of medicines in Member States (Vogler, 2008; Simoens, 2010; Godman et al., 2013a, 2016a; Vogler et al., 2014; Permanand and Pedersen, 2015). Pricing negotiations with pharmaceutical companies are conducted by one or more entities within each Member State. At times, there is lack of trust between different parties (Pharma Diplomacy Working Group, 2016). Concerns with trust are exacerbated by apparently limited correlation between costs for research and development, the costs of producing medicines, their value and the requested prices for new medicines (Experts in CML, 2013; Kantarjian et al., 2013; Paris and Belloni, 2013; Avorn, 2015; Gagnon, 2015; Godman et al., 2015, 2016a; Mailankody and Prasad, 2015; de Bruijn et al., 2016; Hill et al., 2017; Prasad et al., 2017). In view of the different mechanisms, and in spite of external reference pricing, European countries pay different prices for their medicinal products (Kanavos et al., 2011; Leopold et al., 2012, 2013; Vogler et al., 2017).

European health technology initiatives include the development of HTA Networks at a strategic level and the EUnetHTA Joint Action at a technical level. The HTA network was established through Article 15 of Directive 2011/24/EU on the application of patients' rights in cross-border healthcare. The EUnetHTA Joint Action is organized as a voluntary network of national and regional HTA agencies and other HTA organizations involved in pricing and reimbursement. The remit of EUnetHTA includes the production of guidelines and the joint assessment of health technologies. The funding for EUnetHTA Joint Actions is secured up to 2020, and currently the EC is conducting an exercise to study the future for collaboration on HTA following this as part of their new initiative (European Commission Public Health, 2016).

HTA based on evidence and the interpretation of clinical data is designed to support decision making, including consideration of reimbursement when resources are scarce. Overall, there are different tools to support pricing and reimbursement decisions and their monitoring (Paris and Belloni, 2013; Matusewicz et al., 2015). HTA has also utilized multi-criteria decision analysis (MCDA) for analyzing the value of medicines where more than one criterion is relevant (Irwin and Peacock, 2015; Godman et al., 2016a). MCDA is defined as a methodology for appraising alternatives on individual criteria, and combining them into one overall appraisal (Keeney and Raiffa, 1993).

The initiatives among payers to support reasonable pricing of medicines are based on their perceived value, compared to available alternatives, and thereby support the principle of rewarding and incentivizing innovation. However, this requires a clear definition of innovation (Aronson et al., 2012; Ward et al., 2014; Permanand and Pedersen, 2015). Examples of tools that have been developed by payers in association with others to improve pricing deliberations include Value Based Pricing (VBP) and the Transparent Value Framework (TVF; European Commission, 2012; Godman et al., 2015). There is also ongoing research into risk models between payers and providers such as leasing models (Crown et al., 2017). The TVF was developed in response to concerns about the high prices being requested for new orphan medicines and the increasing numbers being made available (Hughes-Wilson et al., 2012; Godman et al., 2015). Similar concerns are expressed about the rising prices of anti-cancer drugs given increasing prevalence rates with limited correlation between clinical benefits and prices (Ghinea et al., 2015; Tefferi et al., 2015; Vivot et al., 2017), leading to suggestions for developing minimum effectiveness criteria for new cancer medicines as well as critiquing existing criteria developed by cancer societies (Ferguson et al., 2000; Kantarjian et al., 2013; WHO, 2015; Wild et al., 2016; Aggarwal et al., 2017). These are based on the fact that many of the new cancer medicines appear to have limited effect on overall survival despite high prices (Kantarjian et al., 2013; Grössmann and Wild, 2017; Salas-Vega et al., 2017). There are ongoing initiatives among cancer groups to improve the valuation of new cancer medicines including the American Society of Clinical Oncology (ASCO), European Society for Medical Oncology (ESMO), Institute for Clinical and Economic Review (ICER), and the National Comprehensive Cancer Network (NCCN; Bentley et al., 2017; Cheng et al., 2017; Shah-Manek et al., 2017). However, there are concerns with some of these (Wild et al., 2016).

Some Member States have seen the need to adopt national initiatives for early access to medicines such as the early access to medicines scheme (EAMS) in the UK (Medicines Healthcare Products Regulatory Agency, 2016; UK, 2017) and the ATU scheme in France (ANSM, 2015). This practice is not shared by all Member States. Pressure is also being put on governments of certain countries to reimburse new medicines in high priority areas such as cancer and those for orphan diseases at high requested prices despite often limited health gain vs. existing medicines (Hughes-Wilson et al., 2012; Simoens et al., 2013; Haycox, 2016; Aggarwal et al., 2017; Grössmann and Wild, 2017). This includes political pressure to introduce the Cancer Drugs Fund in the UK, with recent evidence suggesting no support for ring fencing such monies in the future (Aggarwal et al., 2017). The funding challenges are exacerbated by the often high requested prices for new cancer medicines despite very low manufacturing costs for a number of them (Hill et al., 2017).

Concerns with high prices, combined with the need to achieve value for new medicines, has resulted in a growing number of managed entry agreements (MEAs) among European countries (Ferrario and Kanavos, 2013, 2015). MEAs are arrangements between manufacturers and payers or providers which enable access to health technologies (especially new and expensive technologies) subject to specified conditions. MEAs include a variety of mechanisms addressing uncertainty about the performance of new technologies as well as managing the adoption of technologies in order to maximize their effective use or limiting their budget impact (WHO CCPPRP, 2017). Although, there are more than 10 years of experience with such schemes, there is still limited evidence in support of their effectiveness while there are concerns with a number of their shortcomings including the considerable economic burden associated with a number of the schemes (Adamski et al., 2010; Ferrario and Kanavos, 2013; Permanand and Pedersen, 2015; Garattini and Curto, 2016; Godman et al., 2016a).

### Post-authorization activities

**Figure 1:**
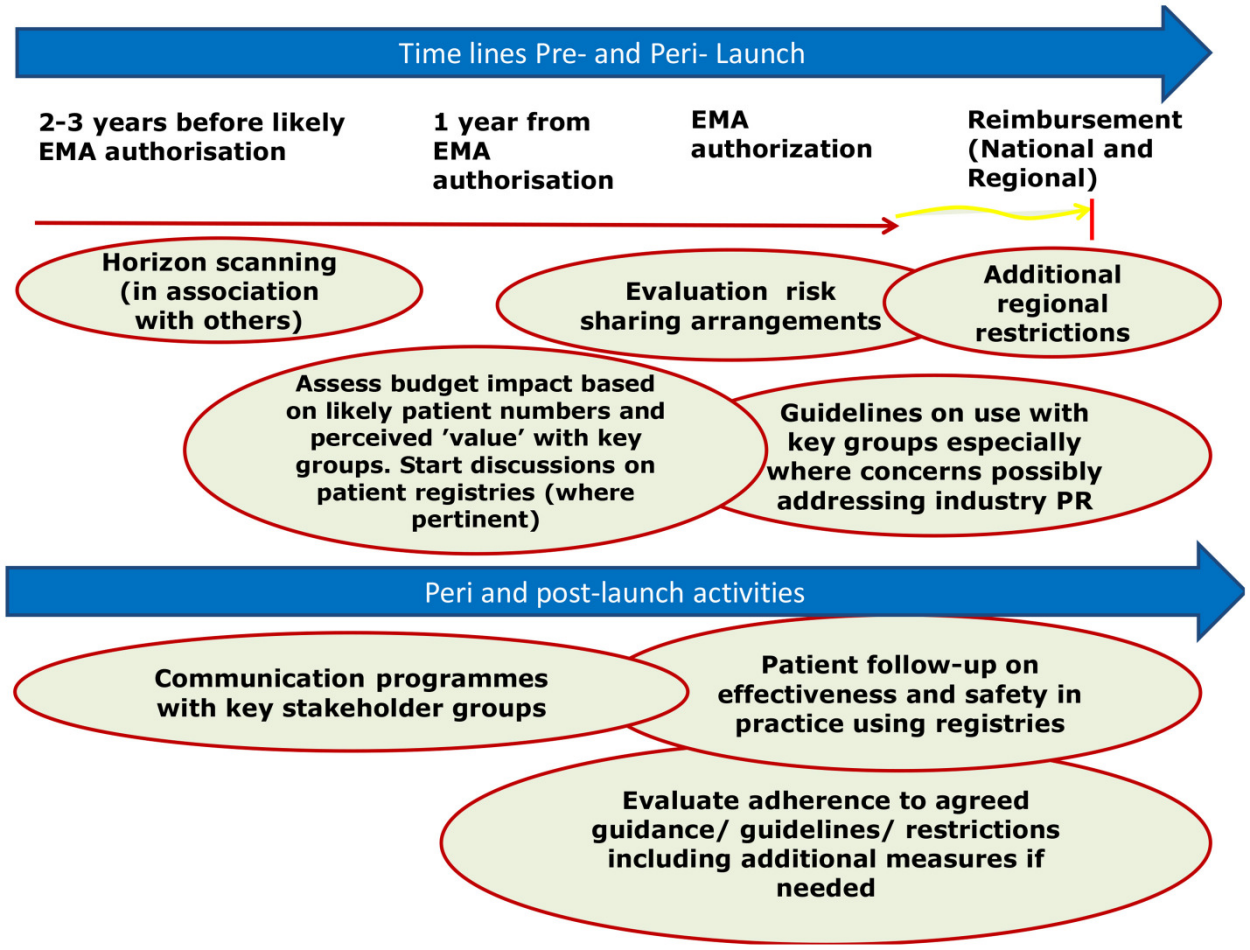
Suggested model to better manage the introduction of new medicines (reproduced with the permission of Frontiers in Pharmacology; Malmstrom et al., 2013).

Once medicinal products are introduced into routine clinical practice, their use is increasingly monitored to ensure prescribing is in line with the recommendations and treatment is optimized (Forslund et al., 2011, 2016; Godman et al., 2014, 2015; Troncoso and Diogene, 2014). This is part of new comprehensive models that have been developed by payers and their advisers across Europe to optimize the managed entry of new medicines (Malmstrom et al., 2013; Godman et al., 2015; Matusewicz et al., 2015; Permanand and Pedersen, 2015; Figure 1). A number of medicinal products, including products with conditional approval and orphan medicinal products, are receiving marketing authorization when there are still uncertainties in their efficacy and safety, and these shift these uncertainties to the post-authorization phase (Joppi et al., 2016).

For innovation to be successful, it should reach the final end user and it should address true unmet need (Kaplan et al., 2013). Patients are the main beneficiaries of innovation as well as being exposed to potential harms in the pharmaceutical framework—this is a concept which may not always be fully appreciated by other stakeholders of the pharmaceutical framework.

### Collaborations and associations to support early access of patients to new medicines

It is recommended by some stakeholders that regulations and HTA be aligned particularly for the requirements of evidence and for considerations for decision making (Bubela et al., 2015). This is reflected by the EMA and some HTA bodies already collaborating on initiatives for parallel EMA-HTA advice to companies. For instance, the Shaping European Early Dialogue (SEED) initiative involves early dialogue organized between developers of pharmaceuticals, European HTA bodies and the EMA (Meyer, 2015; Moseley, 2015).

The EC has also established the Commission Expert Group on Safe and Timely Access to Medicines for Patients (STAMP), which consists of experts from the Member States to provide advice and expertise to the Commission Services in relation to the implementation of EU pharmaceutical legislation and policies and discusses national initiatives and experiences in the field (European Commission, 2017). The EMA has established the Priority Medicines (PRIME) scheme whereby companies with candidate products are given early, proactive and strengthened scientific and regulatory support by the EMA (European Medicines Agency - PRIME, 2017). The Innovative Medicines Initiative (IMI), which is a joint 50/50 initiative between the EC and EFPIA (European Federation of Pharmaceutical Industries and Associations), funds the ADAPT SMART project which involves different stakeholder groups and studies initiatives to support early access of new medicines to patients (ADAPTSMART^8^; ADAPT-SMART^9^).

Most stakeholders (industry, regulatory agencies, health care professionals, patient groups) are represented through groupings and associations. These are generally non-governmental organizations. However, there are only a limited number of payer organizations working together to seek ways to enhance access to new innovative medicines whilst striving to maintain universal access, e.g., the International Association of Mutual Benefit Societies (AIM), the European Social Insurance Platform (ESIP), and the Medicine Evaluation Committee (MEDEV; Schuurman, 2008). The members of these organizations do not always have adequate resources for participation in all initiatives, which potentially impacts their mission.

### Considerations of the concept of adaptive pathways by payers

The introduction of Adaptive Pathways through the regulatory system will impact on the other systems of the pharmaceutical framework including the activities of payers. These changes will include increased uncertainties within the evidence required for HTA given the immaturity of available clinical data at the time of evaluation; increased challenges of conducting budget impact assessments (Sullivan et al., 2014); increased burden, demands and costs for payers in the post-authorization phase; requirements for increased monitoring of the effectiveness and safety of new medicines in routine clinical practice by payers and health service providers during the post-authorization phase and possibly reduced safety and effectiveness of new medicines for patients due to the level of uncertainty (Ermisch et al., 2016). The monitoring burden is enhanced by concerns with available IT systems among member states to robustly collect clinical data in routine care. Increased medicines costs are also a likely threat, with the companies typically seeking higher prices for their new medicines than current standards (Cohen and Felix, 2014; Godman et al., 2015; Howard et al., 2015; de Bruijn et al., 2016).

When medical products receive a conditional marketing authorization by the EMA through the concept of Adaptive Pathways, they will need to proceed through the subsequent systems of the pharmaceutical framework: approval or non-approval by reimbursement/HTA agencies when reviewing and setting prices, approval or non-approval by payers and willingness to adopt or not by healthcare professionals and patients. The evaluation and implementation of the activities related to the adoption of products approved through the concept of Adaptive Pathways will be a challenge especially as some aspects related to Adaptive Pathways present a paradigm shift in the regulatory system. The assurance of risk governance, which is guaranteed through traditional marketing authorization, may be different for medicines authorized through Adaptive Pathways (Ermisch et al., 2016). If new medicines, which attain a traditional marketing authorization, and those with ongoing existing uncertainties about their effectiveness and/ or safety are going to be considered equally authorized, this will increase uncertainty and doubt regarding the safety and effectiveness of all new medicines authorized for patients. We know for instance that the success rate of Phase III studies and submissions is only ~50%, with two thirds of terminations due to lack of efficacy and more than 20% due to safety issues (Ermisch et al., 2016). Consequently, it is of paramount importance to explain the difference between adaptively and conventionally licensed medicines to patients to make sure that they are making informed decisions about their own treatment options. This distinction is seen as essential in order to avoid confusion and to prevent the spill-over of uncertainty surrounding medicines approved through the Adaptive Pathways route to medicines with established benefit/risk safety records. This uncertainty and the lack of assurance offered by marketing authorization may increase the gap and the lack of synchronization and trust between the evaluation for marketing authorization and the technical evaluation during HTA. HTA bodies may increasingly feel the need to redo the evaluation of efficacy from scratch, rather than adopting the efficacy evaluation from the marketing authorization bodies and focusing on the effectiveness evaluation.

The concept of Adaptive Pathways will result in earlier access to patients in routine clinical care only if the new medicines are reimbursed and paid for by payers. Recent experience has shown that in practice the prices for such products will depend on the success of price negotiations, and in some countries a product may end up being prohibitively expensive, or being withdrawn by the manufacturer if they consider the price not satisfactory. Consequently, the goal of early access may not be achieved in practice for a significant proportion of the European population. The decision for reimbursement and the considerations for affordability will be different in the different member states; as a result, the uptake of the medicines will vary between Member States (Putrik et al., 2014). MEAs provide a possibility for consideration of different conditions for reimbursement including risk-sharing agreements and conditions for disinvestment; however, MEAs may not be successful in all countries. Moreover, MEAs are not available in every Member State or may, as mentioned, be considered too costly to administer (Adamski et al., 2010; Ferrario and Kanavos, 2015; Ermisch et al., 2016).

For those countries and health care systems where medicines approved through MAPPs are accepted for reimbursement, and paid for by payers, these medicines will be available to patients. In this case, the HTA evaluation will have to be undertaken with considerable uncertainties and incomplete data. This will result in a greater burden for payers including increased monitoring of the new medicine in routine clinical care, monitoring of prescribing against agreed guidance as well as possibly any added conditions associated with MEAs. It will be unlikely that pharmaceutical companies will bear the costs and the burden for monitoring of treatment, particularly as the medicines will be used within routine clinical practice and not under strict controlled conditions as in Phase III trials. Thus, these uncertainties will be borne by the different systems (including payers and health service providers), and will be experienced down to the patient level. If the burden and the costs for evidence generation for dealing with uncertainties, and for monitoring of safety and effectiveness, which are being shifted from the pre- to the post-authorization phase, will be transferred from pharmaceutical companies onto other stakeholders including payers, these costs will need to be factored into any HTA evaluation and price negotiations. This includes any costs for necessary improvement in IT systems.

The new medicines will need to be monitored for the duration of the conditional approval. Once any new medicine achieves the required evidence, it will be re-evaluated by the EMA and the marketing authorization will be changed to final.

If any one of the processes subsequent to the conditional marketing authorization (pricing, reimbursement, payers, health service providers, and healthcare professionals) does not approve the new medicinal product, the new medicinal product will not be accessible to patients, the ultimate beneficiary. Consequently, for the objective of improved access to be achieved, the perspectives and priorities of all the stakeholders involved need to be met.

## Discussion

Payers will be appreciably impacted by new medicines authorized through MAPPs. The non-binding invitation of the EMA addressing payers to come on board “if relevant” (European Medicines Agency, 2016) is not sufficient to address the key issues raised by payers in their various publications.

Within the current pharmaceutical framework, the main changes are introduced by initiatives arising from individual systems. For example, Adaptive Pathways have been an initiative, a “concept”, introduced and driven by the EMA. Experience has shown that when initiatives are pushed through one system, the other systems which are affected will have to ensure and protect their priorities, their sustainability and their interests. It is important for reimbursement authorities and payers to be alerted and to work on ways to address concerns regarding Adaptive Pathways.

Adaptive Pathways will not necessarily result in improvement in outcomes for patients. Any improvement has to be measured in terms of the benefit to the ultimate beneficiaries, the patients. The introduction of Adaptive Pathways will not address the current issue of delay in access to new products in some Member States due to differences in product launch by marketing authorization holders or due to lack of affordability.

If Adaptive Pathways are implemented, reimbursement authorities and payers may have to make a number of considerations. In reality, only a limited number of new medicines offer a clinical advantage over existing medicines and address unmet medical need (Prescrire Editorial, 2016). There will be a requirement to establish a definition for unmet medical need from the perspective of considering significant gaps in the availability of treatments to patients after full evaluation of alternative treatments which are already available. Secondly, as the new medicines being authorized will have gaps in evidence, and will present appreciably more uncertainty than currently seen with traditional approaches, there will be difficulties in carrying out robust HTA evaluations. The procedure for HTA evaluation for products authorized through Adaptive Pathways should not be distinctive or different from that used for other authorized medicinal products. For legal and equity considerations, in a number of countries it will be considered unacceptable to treat these medicines differently from other authorized medicines. Any procedure dealing with public funding of medicines through Adaptive Pathways must ensure that all medicines are evaluated to the same standards, and if they meet these standards to a different degree, this will be ultimately reflected in their evaluation and reimbursement.

Adaptive Pathways will not support payers to secure good prices for new medicinal products. It seems that in spite of the Treaties and legislation concerning public procurement, within the EU there is no legal framework which demands full transparency and disclosure of costs including R & D costs and cost of goods even when these concern public funds. This lack of transparency does not support the concept of value-based pricing, which seems to be accepted by a number of stakeholders (WHO, 2015; Godman et al., 2016a).

Consideration of opportunity costs could support the achievement of the best use of available resources (Barrett et al., 2006; Haycox, 2016). Decisions including opportunity costs should be managed openly and transparently and involve patients and society. This includes risk-sharing agreements and disinvestment criteria alongside any investment decision made under the Adaptive Pathways framework (Parkinson et al., 2015; Guerra-Júnior et al., 2017). MEAs are likely to become even more important, although, as mentioned, concerns exist including their administrative burden (Adamski et al., 2010; Ferrario and Kanavos, 2013; Ermisch et al., 2016; Garattini and Curto, 2016). In view of this, payers should not accept different conditions for reimbursement for new higher priced medicines authorized via Adaptive Pathways. Differential considerations will be to the detriment of patients whose disease conditions are as relevant but do not happen to be in the same category. Whilst different considerations such as specific funding and less stringent criteria for evaluation are already happening for patients with cancer and orphan diseases in certain countries (Simoens et al., 2013; Cohen and Felix, 2014; Haycox, 2016), further practices of this kind should ideally be avoided to maintain the European ideals of comprehensive and equitable healthcare for all.

Adaptive Pathways will necessarily increase the administrative, logistic and monitoring burden for payers, and these parameters should be transparently factored into any evaluation and costs. This includes any obligations and factors associated with MEAs (Ermisch et al., 2016), including formal disinvestment, should the perceived value of the new medicine not be seen in practice. As medicines approved through Adaptive Pathways will already have a marketing authorization, pricing and reimbursement bodies will need to consider them in line with Council Directive 89/105/EEC^10^ (the Transparency Directive) adding to the burden for payers during this phase where authorization is only provisional.

It will be challenging for payers to stratify the supply of medicines and to restrict the use of these medicines only for the authorized indications. The off-label use of medicines is regulated differently in different countries. It may come with a higher risk for adverse drug reactions (Eguale et al., 2016), and where there is uncertainty related to efficacy and safety, the possibility of risk may be augmented. Another challenge with the implementation of Adaptive Pathways is the collection of safety and effectiveness data when the medicines are used in routine clinical care post-authorization given the limited IT infrastructure in a number of Member States. As these new medicines still have uncertainties in knowledge regarding their effectiveness and safety in the post-authorization phase, they will require additional studies to cover the obligations of the conditional approval. This may require cooperation between pharmaceutical companies and payers to ensure that these obligations are met in a timely and informative manner. There is still a question regarding by whom and how the costs for monitoring, as well as the costs of medicines approved through Adaptive Pathways, will be borne.

Granting high prices for medicines during the collection of efficacy and effectiveness data is not an attractive option for most payers and will be an increasing challenge. Some costs which through the traditional approval would have been borne by pharmaceutical companies in the pre-authorization phase will necessarily shift to the post-authorization phase, and may have to be borne by payers. If such added costs and burden are borne by payers for the whole period until full marketing authorization is granted, difficulties in funding will be increased. The period of conditional approval is not pre-defined and may be long. In view of this, ideally in cases of positive reimbursement decisions by payers, launch prices should take these costs into account. Pharmaceutical companies need to have data on medicines use in clinical practice to fulfill their obligations to give the information required by the regulator to shift these products from conditional approval to full marketing authorization. This data will need to be obtained from health service providers and payers. In exchange, payers may have a stronger position to negotiate with the companies during negotiations on prices and on MEAs. There may also be more financial burden to payers because the uncertainties associated with the effectiveness of these products can result in greater expense if the new medicines are less effective and less safe than expected with more monitoring requirements. In those situations where reimbursement authorities or payers do not consider the reimbursement of medicines authorized through MAPPs positively, pharmaceutical companies may ideally consider paying for the products and supporting their monitoring in order to collate necessary post-authorization data.

Possible collaboration of payers to streamline activities and avoid duplication, and to enhance purchasing and negotiation power building, is recommended. One example of such collaboration is the Beneluxat with Belgium, Netherlands, Luxemburg and Austria collaborating for the management of new medicines (de Block, 2015).

If products are approved through MAPPs, it should be ensured that all stakeholders, including healthcare professionals and patients, are aware of the true uncertainty surrounding these medicines. It should be ensured that adequate provision for liability and informed consent procedures are in place so payers are not additionally burdened by the responsibility for the benefit or risks from the use of these new medicinal products and by any liability costs.

It is also important that research projects and initiatives, which are funded (fully or partially) through EU/public funds, address the objectives of the pharmaceutical framework and include as many different stakeholders as possible to balance interests and perspectives. The current ADAPT SMART project is the main (if not the only) initiative which allows communication between the different stakeholders with regards to Adaptive Pathways. However, some authorities may be hesitant to participate in such initiatives due to concerns that participation in such projects and alliances may compromise their position to take decisions in their best national interest. Nevertheless, these authorities need to express their views publicly—in alternative fora, approaches or projects.

The biggest failure of the pharmaceutical system is that not all patients between and within the Member States have equal access to medicines. This distortion should be addressed first before investing heavily in new, expensive medicines with limited health gain. This is particularly important as most standard medicines are now available as low cost generics, and increasingly as lower cost biosimilars (Woerkom et al., 2012; Matusewicz et al., 2015; Godman et al., 2017).

The principles of subsidiarity and conferral (referring back to the section on Legal and Governance Structure) make a distinction between what is the responsibility of the EC, such as centralized marketing authorization, and what is the jurisdiction of the Member States, such as decisions for pricing and reimbursement. This is a major consideration which helps to secure Member State autonomy and the ability to deal with initiatives and changes emanating from other stakeholders of the pharmaceutical framework. The risk governance and the objectives set by the treaties offered by the current regulatory framework should be secured. This is necessary particularly in view of the lack of alignment, coordination and trust between the different stakeholders of the pharmaceutical framework (Pharma Diplomacy Working Group, 2016). Such uneasiness will be exacerbated by progressing with the Adaptive Pathways process unless it is limited to only a very few truly innovative medicines, with issues of affordability adequately considered. However, the “Public consultation on strengthening EU cooperation on HTA” (European Commission Public Health, 2016) proposes possible changes which may have an impact on the pharmaceutical framework and its various systems.

## Recommendations

Payers need to have plans in place on how to deal with new medicines which will be authorized through the process of Adaptive Pathways. If European payers do not adopt a planned coordinated approach, they run the risk of ending up only reacting to this initiative post factum instead of being in a pro-active position.

New initiatives to change the pharmaceutical framework should aim at supporting increased access to valuable and useful medicines for all patients with true unmet medical need. Such initiatives should be proposed starting from a level playing field for all stakeholders. Increasing equity in the level of coverage for patients should be a primary consideration in the discussions on access and affordability. If the introduction of Adaptive Pathways results in increased shift of burden onto payers, it is unlikely that this will adequately address the differences in the level of availability of medicines to patients across different Member States. Where the shift in burdens and costs result in blocking medicinal products approved through MAPPs to reach patients, pharmaceutical companies should bear or share these costs and burdens. Clear explicit mechanisms to measure the impact on true unmet medical need should be set and acted upon as part of the MAPPs process. Consequently, there needs to be an agreed definition of what constitutes unmet medical need.

There could be different alternatives which allow for out-of-the-box solutions as compared to current accepted practices. Lowering prices may lead to wider use of medicinal products. Currently negotiations between payers and the industry lack transparency especially if confidential discounts as part of MEAs are part of the negotiations. It is not clear whether collaboration between countries is possible for such negotiation procedures, although this is already changing, and collaboration regarding HTA activities is considered positively.

## Conclusion

The example of Adaptive Pathways shows that different systems and stakeholders within the pharmaceutical framework have different priorities and objectives. This renders consensus and strategic collaboration within and between the different systems and stakeholders very difficult.

Clarification is required regarding a number of concerns which have been raised by payers in previous publications. Certain considerations which were not prioritized during the development of the concept of Adaptive Pathways still need to be addressed. To start with, it is important that all players are clear and transparent about the responsibility of the different stakeholders and authorities involved in different processes of the pharmaceutical framework. The minimum level of uncertainty and risk involved should be clear, and the basis for a positive benefit/risk evaluation should be transparent. The regulatory and governance responsibility for products with a centralized marketing authorization legally lies with the EC. Health care professionals and patients need to endorse and clearly accept any additional level of risk which they may share through this new concept.

Payers need to be prepared to deal with new medicines granted conditional approval through Adaptive Pathways. Payers already have experience dealing with conditional approval but need to address the new challenges presented. It is important that in addition to technical considerations, payers should also adopt a wider policy perspective. There are aspects on which payers may benefit from collaborating.

It is likely that the level of accessibility and uptake of new medicines introduced through Adaptive Pathways will be different in different Member States. As experienced with other new medicines, access to new medicines will range from full adoption in some Member States to no access in others. This may depend to some extent on the ability of the legal framework in the individual Member States to accommodate the MAPPs model. The affordability of the products, prioritization and availability of funding are also determinants. Moreover, pharmaceutical companies' willingness to acknowledge the inherent uncertainties of their MAPPs-licensed medicines by accepting lower initial prices, and possibly by fully funding these medicines until full approval, will also play a role. As compared to other medicines introduced for unmet medical need, there may be some shift in the uptake probability curve due to the increased challenges introduced through the regulatory process of Adaptive Pathways. Payers need to meet these challenges in an active way. They need to respect their responsibility toward ensuring equity in the allocation of public resources.

MAPPs should also be closely monitored and followed up to evaluate their actual implementation and their impact on the outcome of addressing the true unmet medical needs of patients in the EU.

Ideally, common agreed outcomes are established for a coordinated strategy for new initiatives aimed to bring about changes to the pharmaceutical framework. A holistic approach for the pharmaceutical framework might provide the basis to obtain a balance between the needs of all stakeholders. Regrettably this is currently not the case. Such coordination will require increased transparency in the way that the different systems operate and affect each other, and increased trust and consideration between stakeholders.

## Author contributions

PV, ME, BG, and ABu helped, developed the concept of the paper and produced the first draft. This was further developed by FA, MvdC, JJ, AS, and RR before all authors, i.e., PV, ME, BG, AM, JV, LB, ABu, FA, ABy, TB, MvdC, ED, IE, JF, MG, IG, MvdG, JG, JJ, RJ, MK, OL, IL, IM, VM, EM, OM, DM, GP, GS, CS, SS, AS, JR, RR, CZ, EZ, and AH critiqued the final draft before submission and re-submission.

### Conflict of interest statement

The majority of the authors are employed by health authorities or health insurance companies or are advisers to them. However, the content of the paper and the conclusions are those of each author and may not necessarily reflect those of the organization that employs them.

## Footnotes

^1^Council Directive 65/65/EEC of 26 January 1965 on the approximation of provisions laid down by Law, Regulation or Administrative Action relating to proprietary medicinal products.

^2^European Commission. Commission Expert Group on Safe and Timely Access to Medicines for Patients “STAMP”. Available online at: https://ec.europa.eu/health/documents/pharmaceutical-committee/stamp_en (Accessed 26.03.2017).

^3^Regulation (EC) No. 726/2004 of the European Parliament and of the Council of 31 March 2004 laying down Community procedures for the authorization and supervision of medicinal products for human and veterinary use and establishing the European Medicines Agency.

^4^Regulation (EC) No. 507/2006 on the conditional marketing authorization for medicinal products for human use falling within the scope of Regulation (EC) No. 726/2004.

^5^Regulation (EC) No. 141/2000 of the European Parliament and of the Council of 16 December 1999 on orphan medicinal products.

^6^Regulation (EC) No. 1901/2006. Regulation (EC) No. 1901/2006 and Regulation (EC) No 1902/2006 of the European Parliament and of the Council of 12 December 2006 on Medicinal Products for Paediatric Use and Amending Regulation (EEC) No. 1768/92, Directive 2001/20/EC, Directive 2001/83/EC and Regulation (EC) No. 726/2004.

^7^Regulation (EU) No. 1235/2010 of the European Parliament and of the Council of 15 December 2010 amending, as regards pharmacovigilance of medicinal products for human use, Regulation (EC) No. 726/2004 laying down Community procedures for the authorisation and supervision of medicinal products for human and veterinary use and establishing a European Medicines Agency, and Regulation (EC) No. 1394/2007 on advanced therapy medicinal products.

^8^ADAPTSMART. Accelerated Development of Appropriate Patient Therapies – a Sustainable Multi-stakeholder Approach from Research to Treatment-outcomes. Available at URL: http://adaptsmart.eu/

^9^ADAPT-SMART. Project Overview. Available at URL: http://adaptsmart.eu/wp-content/uploads/2015/09/ProjectOverview-IMI2-ADAPTSMART.pdf.

^10^Council Directive 89/105/EEC of 21 December 1988 relating to the transparency of measures regulating the prices of medicinal products for human use and their inclusion in the scope of national health insurance systems.
